# Management and outcomes of spontaneous coronary artery dissection: a systematic review of the literature

**DOI:** 10.3389/fcvm.2024.1276521

**Published:** 2024-01-16

**Authors:** Milovan Petrović, Tatjana Miljković, Aleksandra Ilić, Mila Kovačević, Milenko Čanković, Dragana Dabović, Anastazija Stojšić Milosavljević, Snežana Čemerlić Maksimović, Milana Jaraković, Dragica Andrić, Miodrag Golubović, Marija Bjelobrk, Snežana Bjelić, Snežana Tadić, Jelena Slankamenac, Svetlana Apostolović, Vladimir Djurović, Aleksandra Milovančev

**Affiliations:** ^1^ Faculty of Medicine, University of Novi Sad, Novi Sad, Serbia; ^2^ Institute of Cardiovascular Diseases of Vojvodina, Sremska Kamenica, Serbia; ^3^ Faculty of Sport and Physical Education, University of Novi Sad, Novi Sad, Serbia; ^4^ Medical Faculty, University of Niš, Niš, Serbia; ^5^ Clinical Center of Niš, Cardiology Clinic, Niš, Serbia; ^6^ Clinic of Nephrology and Clinical Immunology, University Clinical Center of Vojvodina, Novi Sad, Serbia

**Keywords:** spontaneous coronary artery dissection, treatment, invasive treatment, conservative treatment, outcomes, systematic review

## Abstract

**Background:**

Contemporary management of spontaneous coronary artery dissection (SCAD) is still controversial. This systematic review of the literature aims to explore outcomes in the patients treated with conservative management vs. invasive strategy.

**Methods:**

The PRISMA (Preferred Reporting Items for Systematic Reviews and Meta-Analyses) guidelines were followed when we extensively searched three electronic databases: PubMed, ScienceDirect, and Web of Science, for studies that compared conservative vs. invasive revascularization treatment outcomes for patients with SCAD from 2003 to 2023. The outcomes of interest were all-cause death and major adverse cardiovascular events (MACE), including acute coronary syndrome (ACS), heart failure (HF), need for additional revascularization, target vessel revascularization (TVR), SCAD recurrence, and stroke.

**Results:**

The systematic review included 13 observational studies evaluating 1,801 patients with SCAD. The overall mean age was 49.12 +/− 3.41, and 88% were females. The overall prevalence of arterial hypertension was 33.2%, hyperlipidemia, 26.9%, smoking, 17.8%, and diabetes, 3.9%. Approximately 48.5% of the patients were diagnosed with non-ST elevated myocardial infarction (NSTEMI), 36.8% with ST elevated myocardial infarction (STEMI), 3.41% with unstable angina, 0.56% with stable angina, and 0.11% were diagnosed with various types of arrhythmias. The left anterior descending artery (LAD) was the most common culprit lesion in 51% of the patients. There were initially 65.2% of conservatively treated patients vs. 33.4% that underwent percutaneous coronary intervention (PCI) or 1.28% that underwent coronary artery bypass graft (CABG). SCAD-PCI revascularization was associated with a variable range of PCI failure. The most common complications were hematoma extension and iatrogenic dissection. SCAD-PCI revascularization frequently required three or more stents and had residual areas of dissection. The overall reported in-hospital and follow-up mortality rates were 1.2% and 1.3%, respectively. The follow-up range across studies was 7.3–75.6 months. The authors reported variable prevalence of MACE, recurrent SCAD up to 31%, ACS up to 27.4%, TVR up to 30%, repeat revascularization up to 14.7%, UA up to 13.3%, HF up to 17.4%, and stroke up to 3%.

**Conclusion:**

Our results highlight that conservative treatment should be the preferred method of treatment in patients with SCAD. PCI revascularization is associated with a high prevalence of periprocedural complications. SCAD poses a considerable risk of MACE, mainly associated with TVR, ACS, and recurrent SCAD.

## 1 Introduction

Spontaneous coronary artery dissection (SCAD) is a rare cause of acute coronary syndrome (ACS), typically in patients without classical cardiovascular risk factors (1). The reported incidence of SCAD varies greatly, depending on the methodology and studied cohort. Previous angiographic series have reported a prevalence of SCAD ranging from 0.10% to 0.24% (2–5). Nevertheless, the incidence of SCAD rises particularly among women diagnosed with ACS before the age of 50, exhibiting a reported prevalence of up to 24% (6). Recent studies, mainly national registries, have provided growing data on the pathophysiological features of SCAD, which is now being more recognized as a cause of ACS, particularly among young and middle-aged women. In addition, risk factors for SCAD include pregnancy and peripartum periods, multiparity (i.e., more than three births) (7, 8), fibromuscular dysplasia, connective tissue disorders, hormonal therapy, systemic inflammation, and strong mechanical and emotional stressors (9).

SCAD is defined as a non-traumatic and non-iatrogenic separation of the coronary arterial walls, creating a false lumen (10) between the intima and media or between the media and adventitia. It can potentially arise from an intimal rupture, disrupting the vessel wall, or bleeding in the vasa vasorum, leading to the formation of an intramural hematoma. The false lumen or intramural hematoma might progressively expand as a result of the pressure, leading to increased separation between the dissected layers. This separation can compress the true lumen, resulting in myocardial ischemia or infarction (11). The clinical manifestation of SCAD varies based on the severity and extent of the coronary dissection, encompassing a spectrum from no apparent symptoms to unstable angina, acute myocardial infarction, ventricular arrhythmias, and even sudden cardiac death. Given the association of SCAD with multiple diseases and conditions, it is likely that SCAD represents a diverse and heterogeneous entity (11).

Most SCADs are diagnosed by coronary angiograms. Nevertheless, angiography lacks the ability to visualize the vessel wall and exhibits restricted diagnostic accuracy. However, novel tomographic techniques such as intravascular ultrasound (IVUS), optical coherence tomography (OCT), or multislice computed tomography (MSCT) provide unprecedented diagnostic insights in specific cases (12, 13). Furthermore, MSCT has been used for longitudinal follow-up evaluation of patients with SCAD (11).

Nonetheless, contemporary management is still controversial and it represents the main focus of the research. The management and outcomes of SCAD are substantially different from atherosclerotic ACS. In particular, the question of whether conservative medical management or coronary revascularization offers more benefits and improves outcomes is still unresolved, leaving the matter open to further discussion and research (9, 14). There are no randomized clinical trials that address this research question. Still, the European Society of Cardiology position paper (1) and the American Heart Association Scientific Statement (15) on SCAD favor a conservative strategy when revascularization is not mandatory for hemodynamic instability or ongoing ischemia. This is mostly because of the suboptimal percutaneous coronary intervention (PCI) success and the high risk of peri- and postprocedural complications in the setting of SCAD noted in observational studies (1).

The aim of our systematic review of the literature is to explore outcomes in the patients treated with conservative management vs. invasive strategy.

## 2 Methods

Our systematic review aimed to investigate and compare outcomes of the studies reporting treatment in SCAD patients. Since SCAD is a rare disease and there is a lack of randomized clinical trials comparing treatments, the rationale behind this is the necessity to identify potential treatment recommendations.

### 2.1 Literature search strategy

The PRISMA (Preferred Reporting Items for Systematic Reviews and Meta-Analyses) guideline was adhered to when conducting the systematic review to provide a thorough and transparent report (16). Three electronic databases, PubMed, ScienceDirect, and Web of Science, were searched extensively from 1 January 2003 to 9 May 2023,with the following keywords: “spontaneous coronary artery dissection,” “SCAD,” “coronary artery dissection,” AND “treatment,” “invasive treatment,” “medical treatment,” “conservative therapy,” and “clinical outcomes.” To find other qualified studies that did not turn up in the original search, the lists of references in the eligible articles were also examined. The screening was carried out after the exclusion of duplicate articles. Initially, studies were excluded from the evaluation based on the title and abstract. Studies that were retained for the next phase were then screened and included if they reported any outcomes of invasive or conservative treatment of SCAD. Only articles with an available full-text version were included. Two independent investigators (SM, AM) reviewed all titles and abstracts and selected the potentially eligible ones. Any disagreements between the investigators were resolved by consensus.

### 2.2 Study eligibility

Studies published in the preceding 20 years were considered (with the study period defined as from 1 January 2003 to 9 May 2023). Different types of publications, including books, book reviews, editorials, comments, letters, opinion pieces, reviews, meta-analyses, abstracts from scientific conferences, and case reports were not taken into consideration. For each eligible study, full texts, supplementary materials, and online appendices were examined for inclusion/exclusion criteria.

The inclusion criteria were: original articles published in English, observational or randomized controlled trials, articles that contain or compare two techniques for SCAD treatment (conservative vs. invasive revascularization), and studies that reported outcomes. The outcomes of interest were all-cause death; cardiovascular death; and major adverse cardiovascular events (MACE): acute coronary syndrome (ACS), heart failure (HF), need for additional revascularization, target vessel revascularization (TVR), SCAD recurrence, and stroke. We excluded the following: studies contrasting the two approaches that did not provide clinical results; studies that, despite evaluation of the clinical outcomes, did not report in detail the type of treatment strategy; and studies that were considered very low quality or had inadequate methodology. The original study protocol was registered on the PROSPERO platform with ID CRD42023444058.

### 2.3 Data extraction

**Figure 1:**
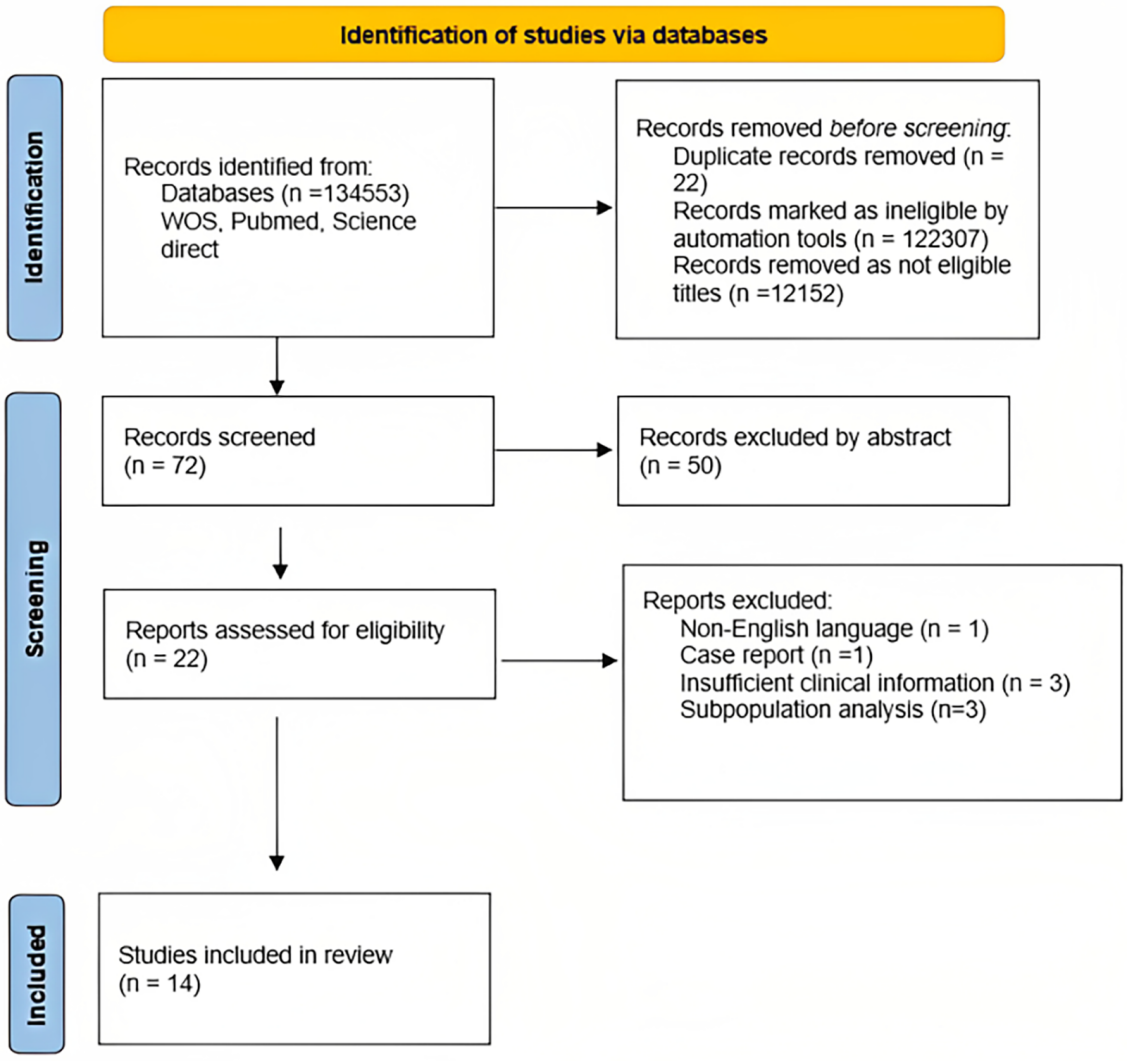
Flowchart PRISMA for management and outcomes of spontaneous coronary artery dissection: a systematic review of the literature.

The key information about the articles included in this review is presented in tabular form (Microsoft Word 2016, Microsoft, Washington, DC, USA), while the analysis of the included literature was performed descriptively. Certain specificities of some studies that go beyond the tabular explanation are described in narrative detail in the Results section. Data regarding study design, sample size, clinical presentation, coronary angiography findings, length of follow-up, and outcomes of interest were extracted from the selected studies. The screening processes have been summarized via the PRISMA flowchart (Figure 1).

### 2.4 Risk of bias assessment

**Figure 2:**
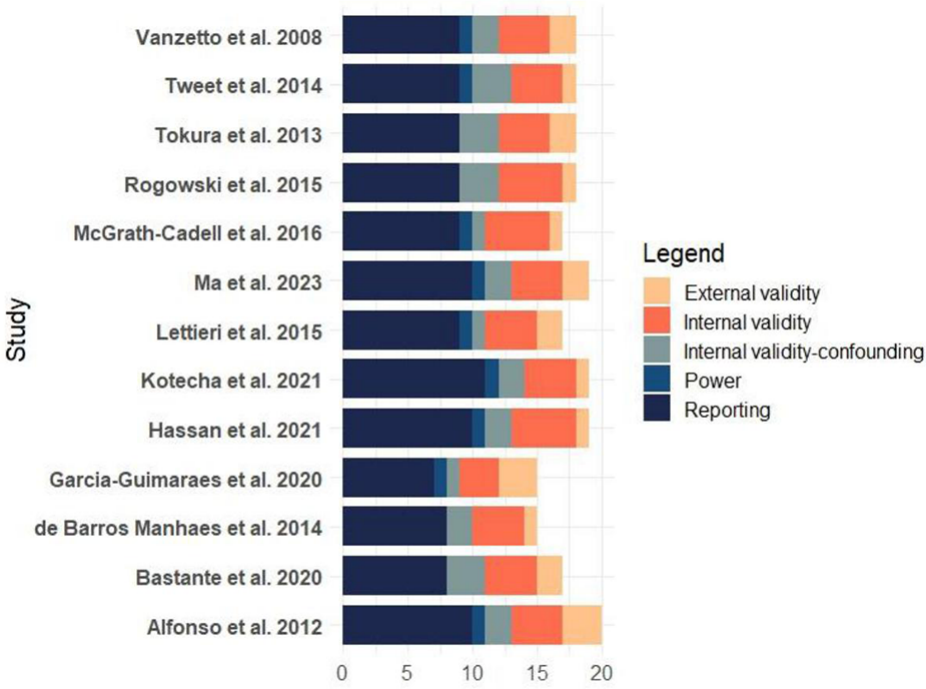
Figure one bias assessment for included studies in the systematic review: management and outcomes of spontaneous coronary artery dissection.

Two independent researchers assessed the risk of bias using the Downs and Black checklist (17). After evaluation, the studies were classified as “high quality” (scoring 23–32), “moderate quality” (score 19–22), “lower quality” (score 16–18), or “poor quality” (score lower than 15) (18). Furthermore, an average of all ratings was generated to estimate the overall quality of the included research. The study design and the Downs and Black scores were used to determine the quality of evidence. Overall, 15.4% of the studies were of poor quality, 53.8% were of low quality, and 30.8% were of moderate quality (Figure 2).

### 2.5 Statistical analysis

Continuous variables are expressed as means ± standard deviations or median (with interquartile range) values, and categorical variables are described as numbers and percentages. To establish inter-rater reliability between two researchers who completed the bias checklist, the interclass correlation statistical approach (SPSS, IBM, New York, USA, v.20) was employed.

## 3 Results

There were 134,553 records identified from databases in the literature search. After removing ineligible records, 72 titles and abstracts were screened, 50 records were removed after abstract reading, and 22 publications were thoroughly assessed according to eligibility exclusion and inclusion criteria. Finally, 13 studies were included in the analysis. All of the studies were observational, with the majority being retrospective and only a few collecting prospective data. There were no randomized clinical studies. The authors reported single-center data in 10 studies, and 3 studies (19–21) were multicentric. Across all studies, sample sizes had a median (range) of 64 (10–436), and 5 trials (38.5%) had a sample size greater than 100.

### 3.1 Presentation and clinical characteristics

The systematic review included 1,801 individuals, and the baseline characteristics are reported in Table 1. Sufficient overall data were available in 13 studies. The overall median age was 49.12 ± 3.41, with 88% being females. Overall prevalence of arterial hypertension was 33.2%, hyperlipidemia, 26.9%, smoking, 17.8%, and diabetes, 3.9%. Out of the total cases, 48.5% were diagnosed with non-ST elevated myocardial infarction (NSTEMI), 36.8% had ST elevated myocardial infarction (STEMI), 3.41% experienced unstable angina (UA), 0.56% had stable angina (SA), and 0.11% were diagnosed with various types of arrhythmias. The left anterior descending artery (LAD) was the most common culprit lesion in 51%, followed by the right coronary artery (RCA) in 24.3%, left circumflex coronary artery (LCX) in 28.1%, and left main coronary artery (LM) in 2.85%. The majority of patients had one vessel disease, but the authors also report multivessel disease in prevalence in up to 13% of the patients (21).

**Table 1:** Basic SCAD patients characteristics.

Author and year	*n*	***F*** (%)	Age	Clinical presentation upon admission (%)					SCAD location (%)				Initial treatment (%)		
				STEMI	NSTEMI	UA	SA	Arrhythmia	LM	LAD	LCX	RCA	CON	CABG	PCI
Vanzzeto et al. (3)	23	74	46 ± 9	30.4	60.9	0	0	8.7	13	52	22	13	43	9	48
Tweet et al. (22)	189	91.5	44 ± 9	37		0	0	0	4	61	25	25	48.7	3.17	46.03
Ma et al. (23)	81	67.9	56.8	23.5	13.6	58	4.9	0	2.5	31.2	9.9	55.6	44.4	6.2	49.4
De Barros Manhaes et al. (25)	25	56	48.8 ± 10	40	40	12	8	0	7.4	48	18.6	25.9	56	4	40
Alfonso et al. (13)	45	58	53 ± 11	40	36	0	9	0	2	53	16	29	80	2.2	17.8
Kotecha et al. (19)	436	93.1	48.5	44.9	47	0	0	0	4.1	61.2	30	20	50.7	0	49.3
Lettieri et al. (27)	134	81	52 ± 11	49.2	40.3	3	0	0	2.8	36.1	14.6	27.1	58.2	3.7	38
McGrath-Cadell et al. (21)	40	95	45 ± 10	30	65	0	0	0	2.5	68	25	18	67.5	5	30
Rogowski et al. (28)	64	94	53 ± 11.2	30	69	0	0	0	4.7	45	45	10	87.5	1.6	10.9
Tokura et al. (24)	10	90	46 ± 17	90		10	0	0	0	40	10	50	10	0	90
Bastante et al. (8)	33	97	56 ± 12	27	73	0	0	0	0	51	24	24	82	0	18
Hassan et al. (29)	403	91.3	48.9 ± 10.1 53.1 ± 9.6	25.6	74.4	0	0	0	1	49.1	32.5	26.1	81.4	0	18.6
Garcia-Guimaraes et al. (20)	318	88	53 ± 13	39	53	2	0	0	2	44	33	21	78	0	22

CABG, coronary artery bypass graft; CON, conservative treatment; F, females; LAD, left anterior descending artery; LM, left main coronary artery; LCX, left circumflex coronary artery; NSTEMI, non-ST elevated myocardial infarction; PCI, percutaneous coronary intervention; RCA, right coronary artery; SA, stable angina; STEMI, ST elevated myocardial infarction; UA, unstable angina.

There were initially 65.2% of conservatively treated patients vs. 33.4% that underwent PCI or 1.28% that underwent coronary artery bypass graft (CABG). The overall rate of PCI conversion into CABG was 3.42%.

### 3.1 Differences between PCI vs. conservative treatment studies

The prevalence of the initial approach varies between studies. Some studies had a similar number of patients treated conservatively vs. revascularization and some favored conservative management. Vanzetto et al. (3) included 23 patients, with conservative treatment in 43% and revascularization in 57%, [CABG in 9% and percutaneous transluminal coronary angioplasty (PTCA) in 48%]. Revascularization procedures were mainly performed in patients with dissection involving theLM and the proximal or mid-LAD, while medical therapy was the preferred strategy in other locations. Tweet et al. (22) reported that those treated with initial revascularization more frequently presented with STEMI compared with those managed conservatively (51% vs. 23%; *p* = 0.0002) with higher rates of vessel occlusion (48% vs. 19%; *p* < 0.0001), larger vessel diameter (2.8 vs. 2.6 mm; *p* = 0.011), and higher mean lesion stenosis (90% vs. 75%; *p *< 0.0001). CABG was performed after PCI failure. Ma et al. (23) divided SCAD patients into high or low risk based on the lesion location and intramural hematoma. LM or proximal coronary artery segment involvement was categorized as high risk. PCI revascularization was the treatment strategy in 49.4%, and in 6.2% CABG was performed compared with the 44.4% managed conservatively. More patients in the high-risk group received PCI (68.4% vs. 32.5%, *p* < 0.01), while most patients in the low-risk SCAD group received conservative management (62.8% vs. 23.7%, *p* < 0.01). Kotecha et al. (19) compared the PCI vs. the conservative treatment cohort. PCI-treated SCAD patients had a higher prevalence of proximal, midvessel, and multisegment coronary artery lesions. Tokura et al. (24) included 10 patients. Thrombus aspiration alone was performed in three patients and it was suggested as one of the possible strategies in selected patients with SCAD, four patients were treated with stenting, two with balloons, and one conservatively.

The subsequent studies preferred initial conservative treatment. The study by de Barros Manhaes et al. (25) included 25 patients predominantly treated medically in 56% of the cases vs. the 40% PCI treated. Only the patient with multivessel dissection was treated with CABG. Alfonso et al. (26) divided SCAD patients into isolated SCAD (60%) and atherosclerosis-associated SCAD (40%). At diagnosis, the initial therapeutic strategy was always conservative medical management. Overall, nine patients (20%) required revascularization for ongoing ischemia at the time of diagnosis, seven were treated with stents, one, with balloon angioplasty, and one with LM SCAD required CABG. The study by Lettieri et al. (27) included 134 patients of which 58% were initially treated conservatively, and 42% underwent coronary revascularization as first-choice therapy. Two patients who were initially treated conservatively underwent subsequent revascularization because of clinical destabilization and angiographic progression of the dissection. CABG was performed for multivessel dissection or left main coronary artery involvement. McGrath-Cadell et al. (21) included 40 patients, of which 68% were managed medically, 30% had PCI, and 5% had CABG (rescue CABG following a ventricular fibrillation cardiac arrest, immediate two-vessel CABG when presenting with LAD and right coronary artery SCADs). Rogowski et al. (28) followed initial conservative strategy in 87.5% (PCI was performed in 9.4% because of impaired flow and ongoing chest pain, and after resuscitation. One urgent CABG was done for LAD and first diagonal branch occlusion). Bastante et al. (8) included 33 patients and initial conservative treatment was the first option in most cases (82%). Only six patients were treated with PCI as the initial strategy, four of them because of progressive flow worsening with contrast injections. The PCI conventional success was reported in 50% of the cases, and the PCI-SCAD success in 67% of the cases. One iatrogenic dissection was reported in the LM.

Hassan et al. (29) included 403 patients, 18.6% underwent PCI of the SCAD-affected artery, and 81.4% were treated conservatively during their initial SCAD hospitalization. Of the 75 SCAD patients who underwent PCI, 60 had PCI as their first-treatment strategy (80.0%), 11 had PCI after failed initial medical treatment (14.6%), and 4 had PCI after thrombolysis (5.3%). PCI was deemed successful in 34.7% (26/75), partially successful in 37.3% (28/75), and unsuccessful in 28.0% (21/75). The indications for PCI were ongoing ischemia, ongoing symptoms, ventricular tachycardia (VT) or ventricular fibrillation (VF), hemodynamic instability, LM dissection, large artery >33 mm, proximal segments, severe stenosis (90%–100%), TIMI 0 or 1 flow, Multivessel SCAD, catheter-induced dissection, and others. Garcia-Guimares et al. (20) included 318 patients. Most patients were initially managed conservatively (78%). Independent predictors of adverse events were initial management with percutaneous coronary intervention (OR, 5.97; 95% CI 1.78–20, *p* = 0.004) and angiographic presentation as intramural hematoma (OR, 4.96; 95% CI 1.19–21; *p* = 0.028).

### 3.2 Medical therapy

Generally, in-hospital medical therapy did not differ from standard pharmacological treatment for patients with ACS. The studies that reported details about medications are narratively described. Alfonso et al. (26) reported that at discharge patients received standard of care dual antiplatelet therapy (DAPT) for up to 12 months, oral anticoagulants, beta-blockers, calcium-channel blockers, and angiotensin system antagonists. In a study by Garcıa-Guimaraes et al. (20), at discharge 92% of patients were on low-dose aspirin and more than half (59%) were on DAPT, although relatively few were on potent antiplatelet agents (ticagrelor, 19% and prasugrel, 3%). Additional treatments at the time of discharge included beta-blockers (79%), statins (79%), and angiotensin-converting enzyme inhibitors or angiotensin II receptor blockers (ACEi/ARB) (51%). Other authors (25) also reported that the pharmacological therapy was based on the combination of antithrombotic and anti-ischemic drugs. Lettieri et al. (27) reported 94% of PCI patients and 82% of medically treated patients received DAPT. Ticlopidine, clopidogrel, and a new P2Y12 receptor inhibitor were used in 6%, 87%, and 7% of the patients, respectively, whereas 6% received oral anticoagulants. DAPT was continued for 11.9 ± 7.1 months. A group of multicenter Australian authors in their multicentric study reported (21) that among medically managed patients, 78% were prescribed a beta-blocker, 89%, aspirin, 74%, an additional antiplatelet agent, 59%, an ACEi/ARB, 41%, a statin, and 7%, a calcium channel blocker. Rogowski et al. (28) reported that aspirin was prescribed in 97%, DAPT in 92%, oral anticoagulants in 6%, statin in 89%, beta-blockers in 86%, ACEi/ARB in 36%, and calcium channel blockers in 19% of the cases. Bastante et al. (8) reported prescribed ASA in 94%, clopidogrel in 27%, ticagrelor in 15%, DAPT in 42%, anticoagulation in 6%, beta-blockers in 85%, ACEi/ARB in 64%, statins in 76%, nitrates in 9%, and Ca-channel blockers in 9%.

### 3.3 Characteristics of SCAD revascularization

In the cohorts that were treated by PCI, variable incidences of complications were reported. Alfonso et al. (26) reported PCI-associated complications in 25% of the patients in the PCI group vs. the 2.7% of the conservative group. The catheter-induced, remote iatrogenic dissection of the vessel initially treated with stents underwent a second intervention with additional stent implantation in segments showing severe residual dissections. Venzeto et al. (3) reported PTCA failure in 27.2% of the patients with immediate or delayed (<48 h) extension of the dissection requiring emergency CABG. Tweet et al. (22) reported PCI failure occurrence in 53% overall, while when SCAD specific criteria (flow-based) were used, the failure rate was 30%. The reasons for technical failure in the PCI group with preserved vessel flow (23/46) were failure to cross the vessel with a wire or device because of wire entry into a false lumen (7/23), and final loss of flow after stent placement with residual stenosis >30%. Hassan et al. (29) reported PCI as successful in 34.7%, partially successful in 37.3%, and unsuccessful in 28%. The majority of the PCI-treated patients (73.3%) had stent implantation (5/55 were unsuccessful), angioplasty alone was performed in 16% (8/12 cases were unsuccessful), wiring alone was attempted in 10.7% (8/8 were unsuccessful), and cutting balloon was used in only one case. The mean number of stents implanted was 2.6 ± 1.8, and more than three stents were used in 15% of the cases. Of all PCI cases, propagation of SCAD occurred in 44%, and residual dissection was observed in 58.7%. Final TIMI 3 flow was observed in 72%, and improved TIMI flow with PCI occurred in 62.7%. Four patients required emergency bailout CABG (5.3%). In the study by Kotecha et al. (19) with the highest cohort of PCI patients (215), 72.6% of the patients underwent stenting, mostly with drug-eluting stents, 20.9% had balloon angioplasty, 5%, with cutting balloons, and 6.5% underwent wiring only. The mean number of stents deployed was 2.3 (range 1–8) per stented case, 10.6% of all SCAD-PCI cases required four or more stents. The median total length of deployed stents was 46 mm, with 29.8% of all SCAD-PCI cases requiring ≥50 mm stents, and 64.1% of the stented cases were left with residual unstented areas of dissection. PCI complications occurred in 38.6% of SCAD-PCI patients with the most common being hematoma extension in 27% and iatrogenic dissection in 8.4%. The total number of implanted stents emerged as a positive predictor for the risk of complications (OR = 1.90; 95% CI 1.26–2.85), and the maximum stent diameter remained associated with risk of serious complications in SCAD-PCI patients (OR = 2.62; 95% CI 1.28–5.39). Lettieri et al. (27) reported procedural success of PCI in 72.5% of the patients. Initial PCI was unsuccessful in 5.9%. In the study that included 318 patients, Garcıa-Guimaraes M et al. (20) found that the most common PCI procedures were drug-eluting stent implantation (58%), simple balloon angioplasty (15%), and bioresorbable device implantation (13%). The PCI success rate was 57% according to the conventional definition and 81% according to the SCAD-specific definition. Similar PCI conventional success (50%) was reported by Bastante et al. (8), and PCI-SCAD success was observed in 67% of the cases.

Tokura et al. (24) reported PCI protocol on 10 patients, where first aspiration thrombectomy was tried, then if sufficient blood flow was not obtained, subsequently balloon angioplasty followed, and bare metal stents were placed as the final step.

### 3.4 Outcomes

Short- and long-term outcomes are reported in detail in Table 2. In-hospital MACE prevalence was low. Overall reported in-hospital mortality was also low (1.2%). In-hospital mortality was reported in both groups in 12 studies, in total eight deaths in the conservative treatment group and seven in the revascularization group; the others did not classify mortality based on the treatment group. The follow-up (FU) range across studies was 7.3–75.6 months. Median mortality rate in FU was 1.3% (2.7% conservative vs. 2.5% revascularization group). Event-free follow-up MACE was high from 74% to 94%. The authors reported variable prevalence of follow-up MACE, recurrent SCAD up to 31%, ACS up to 27.4%, TVR up to 30%, repeated revascularization up to 14.7%, UA up to 13.3%, HF up to 17.4%, and stroke up to 3%. It is possible to explain the variation in the occurrence of MACE in different studies based on the number of patients included and the initial treatment approach. The observational nature of some studies could lead to selection bias and result in varying frequencies of MACE. Nevertheless, studies that included the highest number of patients treated with PCI overall showed a higher prevalence of MACE. In addition, in studies with the highest number of included patients (20, 29), PCI was deemed as suboptimal. The general conclusion in the majority of included studies is that conservative treatment should be the preferred method of treatment. In studies that did report follow-up angiographies, a high prevalence of vessel healing in bout groups was observed (23).

**Table 2:** Outcomes and follow-up of included SCAD studies patients.

Author and year	In-hospital death (%)	In-hospital MACE (%)	Median FU time	FU mortality (%)	FU MACE (%)	Cross-over during hospitalization	Major outcomes
Vanzzeto et al. (3)	8.7	NR	15.6 months	Overall 4.5	HF: 17.4	3 PCI → CABG	1-year event-free survival was 74%
Tweet et al. (22)	0.5 REV	NR	2.3 years	Overall: 2 1 REV vs. 4 CON	Recurrent SCAD: 23 REV vs. 31 CON TVR: 30 REV vs. 19 CON HF: 12 REV vs. 16 CON	13 PCI → CABG 9 CON → CABG / PCI	PCI for SCAD is associated with significant rates of complications and urgent CABG. CABG offers excellent early outcomes for certain patients. The risk of long-term TVR or recurrent SCAD is not decreased by revascularization
Ma et al. (23)	0	NR	1 year	1.2 PCI	Overall: 12.3 UA: 5 REV (PCI) vs. 8.3 CON HF: 5.6% CON	NR	Rates of vessel healing are comparable in CON (low-risk SCAD) vs. REV (high-risk SCAD) group
De Barros Manhaes et al. (25)	0	Overall: 8 Stroke: 4 AMI: 4	75.6 ± 43.1 months	Overall 5.3	5.3 ACS	0	In-hospital MACE-free event was 92%, one patient in the CON group had stroke and one in the PCI group had recurrent AMI. In long FU, 84.2% event-free rate was reported
Alfonso et al. (26)	2.2	0	730 days	0	Overall: 6.6 (all in PCI) HF: 2.2 TVR: 4.4	7 CON → PCI 1 → CABG	At 3 years, 94% and 88% of patients in the I-SCAD and A-SCAD groups, respectively, were free of adverse events
Kotecha et al. (19)	0	NR	900 days	Overall: 0.9 (1.4 PCI vs. 0.5 CON)	Overall: 12.1 REV: PCI 14.4 vs. 9.5 CON ACS: 9.3 PCI vs. 7.7 CON TVR: 4.7 PCI vs. 1.4 CON Stroke: 1.5 PCI vs. 0.7 CON Re-SCAD: 6.1 PCI vs. 6.8 CON	2 PCI → CABG 2 CON → CABG	There was no difference in MACE events between SCAD-PCI and SCAD-non-PCI patients. Although more extensive stenting may be required, with an elevated risk of procedural complications, improved coronary flow and good medium-term outcomes can be achieved with PCI
Lettieri et al. (27)	Overall: 2.2 (1.3 CON vs. 3.6 REV)	AMI 5.2 (2.6 CON vs. 8.9 PCI)	22 months	Overall: 3.1 (2.7 CON vs. 3.8 REV)	Stent thrombosis: 2.4 REV ACS: 1.3 CON vs. 1.9 REV HF: 4 CON vs. 3.8 REV Repeated re-vascularization: 1.3 CON vs. 9.4 REV	2 CON → PCI 3 PCI → CABG 5 re-PCI→CABG	The prognosis in the short and long term in the CON and REV group is generally good, PCI procedure success was less than anticipated and case-specific treatment is manageable and safe
McGrath-Cadell et al. (21)	0	NR	16 months	0	ACS: 10 (7.5 recurrent SCAD in CON; 2.5 stent thrombosis in PCI) Coronary artery aneurysm: 5	3 PCI → CABG	13% of patients had multiple coronary areas involved. The major associated vascular condition is FMD
Rogowski et al. (28)	1.6 (PCI)	NR	4.5 years	0	ACS: 3.2 (CON) Persistent dissection:1.1 (CON)	3 CON → PCI	The long-term results are favorable with conservative treatment
Tokura et al. (24)	0	NR	7.3 months	0	Recurrent SCAD: 10	0	Regardless of the initial treatment, hospital mortality is low, but PCI is linked to a high prevalence of complications
Bastante et al. (8)	0	15 (CON)	33 months	Overall 6.1	Overall: 18 In stent restenosis 3 (PCI) Recurrent SCAD: 12 Chest pain: 9.1 ACS: 9 HF: 3 Stroke: 3	NR	Favorable outcomes were observed in CON vs. PCI. In CON, utilizing a low-intensity antithrombotic strategy using only ASA, and for a limited duration, appears to yield favorable outcomes
Hassan et al. (29)	0.3	Overall: 29.3 PCI vs. 2.8 CON AMI: 20 PCI vs. 1.2 CON Re-revascularization: 18.7 PCI vs. 0.9 CON CVA: 4 PCI vs. 0.6 CON	3.7 years	1.2 non-PCI	Overall: 58.7 (PCI) vs. 22.6 CON Post-discharge MACE: 24 PCI vs.19.8 CON UA: 13.3 PCI vs. 5.8 CON Recurrent AMI: 17 PCI vs. 18 CON Recurrent *de novo* SCAD: 6.7 PCI vs. 12.2 CON Repeat revascularization: 14.7 PCI vs. 3 CON CVA: 1.3 PCI vs. 1.2 CON	11 CON → PCI	In comparison with conservative therapy, PCI was linked to worse procedural success, increased hospital complications from recurrent MI, repeat revascularization, and stroke, as well as long-term risk from repeat revascularization
Garcia-Guimaraes et al. (20)	Overall 1.3	Reinfarction: 3 Unplanned revascularization: 4 Stroke: 1 HF: 1	NR	NR	TVR: 1.3	8 CON → PCI	Most patients were initially treated with a conservative approach and survival rates from admission to discharge were excellent. Outcomes of PCI as first-line therapy were suboptimal

CON, conservative treatment group; REV, revascularization group; PCI, percutaneous coronary intervention; TVR, target vessel revascularization; CABG, coronary artery bypass grafting; A-SCAD, SCAD associated with coronary artery disease; I-SCAD, “Isolated” SCAD; MACE, major adverse cardiovascular event; AMI, acute myocardial infarction; ACS, acute coronary syndrome; FMD, fibromuscular dysplasia; IMH, intramural hematoma; HF, heart failure; CVA, cerebrovascular accident; ASA, acetylsalicylic acid; LM, left main coronary artery; LAD, left anterior descending artery; NR, not reported.

## 4 Discussion

Our systematic review of the literature tried to explore outcomes in patients diagnosed with SCAD treated with either conservative management or invasive strategy. Generally, authors reported that revascularization with PCI is associated with a higher prevalence of complications. Overall short- and long-term mortality is low and irrelevant to treatment strategy. Furthermore, event-free rate for follow-up is high. The most often reported MACE in FU was TVR, recurrent SCAD, and ACS. CABG offers excellent results in particular cases.

### 4.1 SCAD population characteristics

In our systematic report overall, median age was 49.12 +/− 3.41, with 88% being females. Our results are complementary with other systematic reviews and meta-analyses (9, 14, 30) that reported that SCAD primarily affects young- to middle-aged women. Although there are limited data available on SCAD due to its rarity, studies have consistently shown that women, particularly those between the ages of 30 and 50 years, represent the majority of the cases. However, it is important to note that SCAD can affect people of any age and gender. The age range of reported SCAD cases extends from 18 to 84 years, highlighting the variability in its occurrence. The reasons behind the higher prevalence of SCAD in young- to middle-aged women are still not fully understood. Some potential contributing factors include hormonal changes, such as those occurring during pregnancy or in the postpartum period, as well as underlying connective tissue disorders. Emotional stress and extreme physical exertion have also been implicated as potential triggers for SCAD in some cases. We observed a low prevalence of traditional atherosclerotic risk factors in studies included in our review. Our results were very similar to Clare et al. who compared a cohort of 208 SCAD patients with other patients presenting with ACS and found much lower prevalence of atherosclerotic risk factors, as follows: hypertension (30.8% vs. 64.8%), hyperlipidemia (27.9% vs. 62.2%), obesity (18.7% vs. 21.1%), diabetes mellitus (8.2% vs. 35.6%), and chronic kidney disease (4.3% vs. 24.3%). It is known that SCAD patients have fewer traditional risk factors (22). Nonetheless, contrary to the prior understanding, many patients do pose risk factors for ischemic heart disease, such as hypertension, smoking, and dyslipidemia, though there is no firm evidence that these contribute directly to the increased risk of SCAD (1).

In patients presenting with ACS, SCAD is generally rare, approximately noted to occur in 3%–4% (13). Among women presenting with ACS, the prevalence was reported to be higher, at 8.7% in those under 50 years old (3). Some authors reported a much higher prevalence of 24% (10) and 35% (31) in women <50 years after reviewing angiographies, thus highlighting that diagnosis can be often missed. The clinical presentation of patients with SCAD can vary widely. In our systematic review, the majority of patients, 84%, presented with ACS, with a higher prevalence of NSTEMI, compared with STEMI. In two previous studies, it was found that a greater proportion of patients with SCAD presented with STEMI ranging from 80% to 84%, in contrast to NSTEMI, which accounted for only 8%–16% of cases, while 4% of patients presented with UA (32, 33). The reason could be a lower prevalence of angiographies in NSTEMI in the previous reports. In various series, the reported prevalence of SCAD cases presenting with STEMI range from 26% to 87%, in addition, 13%–69% of SCAD patients present with NSTEMI (10, 27, 28, 31). Chest pain is usually the most commonly reported symptom (24) in 95% of the cases (15), though there are reported cases of shock (3) and sudden cardiac death (24). Nearly half of the patients with SCAD commonly had the LAD identified as the most frequent culprit lesion in our report. Similar observations have been reported in other study groups as well (34, 35).

### 4.2 SCAD prognosis and treatment

In recent years, there has been a notable rise in the diagnosis of SCAD owing to the increased awareness among cardiologists and the advancements in intracoronary imaging techniques. Nevertheless, optimal SCAD treatment is still debated because of the lack of large-series registries and randomized trials. The mechanism of vessel obstruction, the acute response of the blood vessel to balloon dilation, and the natural progression of conservatively managed lesions exhibit notable differences between SCAD and atherosclerotic ACS. In SCAD, vessel obstruction occurs as a result of a tear or separation within the layers of the coronary artery wall, forming a false lumen. This mechanism differs from the gradual buildup of plaque seen in atherosclerosis, which is the primary cause of vessel obstruction in ACS of an atherosclerotic origin. When it comes to the acute response of the blood vessel during balloon dilation, SCAD and atherosclerotic ACS again display dissimilarities. The characteristics of the affected vessel, such as its fragility and susceptibility to further dissection, influence the response to balloon dilation in SCAD cases. On the other hand, in atherosclerotic ACS, balloon dilation is typically employed to address the plaque and restore blood flow through the narrowed artery. Furthermore, the natural history of conservatively managed lesions also varies between SCAD and atherosclerotic ACS. SCAD lesions may exhibit a propensity for spontaneous healing and resolution over time in 70%–97% (22, 28, 36) of the selectively restudied patients after a conservatively managed index episode, whereas atherosclerotic lesions often require long-term medical management to prevent disease progression and subsequent complications. Angiographic healing is usually observed within a month (15).

The summarized data in our study suggest that conservative treatment should be absolutely preferred for patients with SCAD. In addition to this, the existing evidence strongly supports (7, 15) the adoption of a conservative approach in the majority of cases, reserving PCI with stent implantation only for unstable patients with compromised distal flow and evident ongoing ischemia. In conservatively treated patients, adverse events are usually seen in the first 7 days, hence intensive monitoring is recommended during this time. There is substantial evidence that the majority of SCAD cases tend to stabilize and eventually heal entirely over time when managed conservatively (1).

Thrombolysis is not recommended for the immediate treatment of SCAD, anticoagulants are also not recommended in routine use (1). The use and duration of antiplatelet therapies for SCAD patients are subjects of debate and vary among reported studies. As there are no randomized trials that investigate the risks and benefits of particular pharmacological treatments, recommendations are based on observational studies and available data. For patients who undergo stenting, current ACS guidelines suggest dual antiplatelet therapy for 12 months, followed by prolonged or lifelong monotherapy (usually with low-dose aspirin). Many experts support the use of acute DAPT during the initial phase, typically involving aspirin and clopidogrel rather than newer P2Y12 inhibitors while avoiding intravenous antiplatelet therapies (15). While DAPT has been widely used in SCAD, there is some registry evidence that DAPT in conservatively treated patients is associated with a significantly higher incidence of MACE when compared with single antiplatelet therapy. DAPT has been prescribed for 12 months in almost all cases (37).

Currently, a prescription of beta-blocker in SCAD is considered for left ventricular dysfunction, arrhythmias, or other indications in SCAD patients (15). Nonetheless, Saw et al. (6) reported that beta-blockers appeared to be protective in long-term cardiovascular events. As there are no randomized controlled trials to support the evidence of different treatment regimes in SCAD, the newly released Scientific Statement of the American Heart Association strongly endorsed the need for multidisciplinary and international trials for SCAD treatment. Alfonso et al. (38) published a protocol of randomized trial that will assess the efficacy and safety of different antiplatelet and beta-blocker regimes for SCAD patients. It is expected that the study will be finished in September 2024. Statins are not routinely advised for SCAD patients. There was conflicting evidence of statin safety and efficiency in SCAD (38). Decisions on other pharmacological regime prescriptions like AC inhibitors should be guideline based and individualized (1). Overall, the management of SCAD patients involves complex decisions and may vary based on individual circumstances and guidelines.

### 4.3 Outcomes

Our study reports low in-hospital and long-term mortality, of 1.2% and 1.3% retrospectively, on 1,801 SCAD patients. Bocchino et al. reported all-cause deaths in 2.9% of patients in the medical treatment group and 4.8% of patients in the revascularization group during a mean follow-up of 28 ± 14 months, without significant differences pooled from 24 studies. In addition to these, meta-analysis reported mortality rates without statistical difference between the two treatment approaches (14). SCAD-associated ACS is linked to a more favorable prognosis compared with atherosclerotic coronary artery disease (35). Nevertheless, we reported that SCAD poses a significant risk of MACE, mainly associated with TVR, ACS, and recurrent SCAD. Similar results are also reported by Martins et al. (14), where a significantly higher risk of TVR was found in the PCI-treated group. Recurrence of SCAD is also widely observed (9).

## 5 Conclusion

The aggregated information within our study indicates that SCAD is more prevalent among females with a low occurrence of traditional atherosclerotic risk factors. This systematic search review provided summarized data from similar studies that compared treatment strategies and outcomes. Our findings suggest that conservative treatment should be absolutely preferred in patients with SCAD, as PCI revascularization is linked to a higher prevalence of periprocedural complications. Revascularization benefits are not widely confirmed, thus it should be the treatment option for high-risk patients with hemodynamic instability, ongoing ischemia, and LM artery involvement. The medical approach to treating SCAD involves using beta-blockers if there are no contraindications, considering DAPT for a duration that ranges from 1 to 12 months (which remains a subject of debate), prescribing statins if atherosclerosis is present, and avoiding anticoagulants that could potentially worsen the expansion of an intramural hematoma (the primary cause of ischemia-induced SCAD). The overall short- and long-term mortality rates for SCAD are generally low and independent of the treatment strategy. Expected MACE prevalence is high in the SCAD population and it is reported in up to one third of patients with TVR, recurrent SCAD and ACS being the most frequent events. These findings could help us derive clinical decisions on a daily basis, likely reducing morbidity and mortality in this rare disease.

## References

[1] Adlam D Alfonso F Maas A Vrints C, Writing Committee. European Society of Cardiology, acute cardiovascular care association, SCAD study group: a position paper on spontaneous coronary artery dissection. Eur Heart J. (2018) 39(36):3353–68. 10.1093/eurheartj/ehy080 29481627 PMC6148526

[2] Ahmed MA Hamra M Ali M Abdullah AS Arnous S Kiernan TJ. Spontaneous coronary artery dissection, challenges of diagnosis and management. Futur Cardiol. (2017) 13(6):539–49. 10.2217/fca-2017-0050 29064286

[3] Vanzetto G Berger-Coz E Barone-Rochette G Chavanon O Bouvaist H Hacini R Prevalence, therapeutic management and medium-term prognosis of spontaneous coronary artery dissection: results from a database of 11,605 patients. Eur J Cardiothorac Surg. (2009) 35(2):250–4. 10.1016/j.ejcts.2008.10.023 19046896

[4] Tsimikas S Giordano FJ Tarazi RY Beyer RW. Spontaneous coronary artery dissection in patients with renal transplantation. J Invasive Cardiol. (1999) 11(5):316–21. 10745540

[5] Hering D Piper C Hohmann C Schultheiss HP Horstkotte D. Prospective study of the incidence, pathogenesis and therapy of spontaneous, by coronary angiography diagnosed coronary artery dissection. Z Kardiol. (1998) 87(12):961–70. 10.1007/s003920050253 10025069

[6] Saw J Starovoytov A Aymong E Inohara T Alfadhel M McAlister C Canadian spontaneous coronary artery dissection cohort study: 3-year outcomes. J Am Coll Cardiol. (2022) 80(17):1585–97. 10.1016/j.jacc.2022.08.759 36265953

[7] Motreff P Souteyrand G Dauphin C Eschalier R Cassagnes J Lusson JR. Management of spontaneous coronary artery dissection: review of the literature and discussion based on a series of 12 young women with acute coronary syndrome. Cardiology. (2010) 115(1):10–8. 10.1159/000244608 19816020

[8] Bastante T Garcia-Guimaraes M Muniz M Cuesta J Rivero F Antuna P Contemporary management of spontaneous coronary dissection. REC-Interv Cardiol. (2020) 2(4):247–55. 10.24875/RECICE.M20000096

[9] Bocchino PP Angelini F Franchin L D’Ascenzo F Fortuni F De Filippo O Invasive versus conservative management in spontaneous coronary artery dissection: a meta-analysis and meta-regression study. Hellenic J Cardiol. (2021) 62(4):297–303. 10.1016/j.hjc.2021.02.013 33689856

[10] Saw J. Spontaneous coronary artery dissection. Can J Cardiol. (2013) 29(9):1027–33. 10.1016/j.cjca.2012.12.018 23498840

[11] Vrints CJM. Spontaneous coronary artery dissection. Heart. (2010) 96(10):801–8. 10.1136/hrt.2008.162073 20448134

[12] Maehara A Mintz GS Castagna MT Pichard AD Satler LF Waksman R Intravascular ultrasound assessment of spontaneous coronary artery dissection. Am J Cardiol. (2002) 89(4):466–8. 10.1016/S0002-9149(01)02272-X 11835932

[13] Alfonso F Paulo M Gonzalo N Dutary J Jimenez-Quevedo P Lennie V Diagnosis of spontaneous coronary artery dissection by optical coherence tomography. J Am Coll Cardiol. (2012) 59(12):1073–9. 10.1016/j.jacc.2011.08.082 22421300

[14] Martins JL Afreixo V Santos L Costa M Santos J Goncalves L. Medical treatment or revascularisation as the best approach for spontaneous coronary artery dissection: a systematic review and meta-analysis. Eur Heart J-Acute Cardiovasc Care. (2018) 7(7):614–23. 10.1177/2048872617706502 28452228

[15] Hayes SN Kim ESH Saw J Adlam D Arslanian-Engoren C Economy KE Spontaneous coronary artery dissection: current state of the science: a scientific statement from the American Heart Association. Circulation. (2018) 137(19):e523–57. 10.1161/CIR.0000000000000564 29472380 PMC5957087

[16] Page MJ McKenzie JE Bossuyt PM Boutron I Hoffmann TC Mulrow CD The PRISMA 2020 statement: an updated guideline for reporting systematic reviews. Br Med J. (2021) 372:n71. 10.1136/bmj.n71 33782057 PMC8005924

[17] Downs SH Black N. The feasibility of creating a checklist for the assessment of the methodological quality both of randomised and non-randomised studies of health care interventions. J Epidemiol Community Health. (1998) 52(6):377–84. 10.1136/jech.52.6.377 9764259 PMC1756728

[18] Tremblay MS LeBlanc AG Kho ME Saunders TJ Larouche R Colley RC Systematic review of sedentary behaviour and health indicators in school-aged children and youth. Int J Behav Nutr Phys Act. (2011) 8:98. 10.1186/1479-5868-8-98 21936895 PMC3186735

[19] Kotecha D Garcia-Guimaraes M Premawardhana D Pellegrini D Oliver-Williams C Bountziouka V Risks and benefits of percutaneous coronary intervention in spontaneous coronary artery dissection. Heart. (2021) 107(17):1398–406. 10.1136/heartjnl-2020-318914 34006503 PMC8372386

[20] Garcia-Guimaraes M Bastante T Macaya F Roura G Sanz R Barahona Alvarado JC Spontaneous coronary artery dissection in Spain: clinical and angiographic characteristics, management, and in-hospital events. Rev Esp Cardiol. (2021) 74(1):15–23. 10.1016/j.recesp.2020.02.008 32418854

[21] McGrath-Cadell L McKenzie P Emmanuel S Muller DWM Graham RM Holloway CJ. Outcomes of patients with spontaneous coronary artery dissection. Open Heart. (2016) 3(2):e000491. 10.1136/openhrt-2016-000491 27621835 PMC5013459

[22] Tweet MS Eleid MF Best PJM Lennon RJ Lerman A Rihal CS Spontaneous coronary artery dissection revascularization versus conservative therapy. Circ-Cardiovasc Interv. (2014) 7(6):777–86. 10.1161/CIRCINTERVENTIONS.114.001659 25406203

[23] Ma Y Zhong X Yin J Lu H Pan C Huang D Treatment strategy for spontaneous coronary artery dissection based on anatomical characteristics. Eur J Med Res. (2023) 28(1):29. 10.1186/s40001-023-00986-y 36647158 PMC9841636

[24] Tokura M Taguchi I Kageyama M Nasuno T Nishiyama Y Koshiji N Clinical features of spontaneous coronary artery dissection. J Cardiol. (2014) 63(2):119–22. 10.1016/j.jjcc.2013.07.001 24012329

[25] de Barros Manhaes E Gomes WF Bezerra CG Horta PE da Gama MN Cesar LAM Spontaneous coronary artery dissection: therapeutic approach and outcomes of a consecutive series of cases. Rev Bras Cardiol Invasiva. (2014) 22(1):32–5. 10.1016/S2214-1235(15)30176-9

[26] Alfonso F Paulo M Lennie V Dutary J Bernardo E Jiménez-Quevedo P Spontaneous coronary artery dissection: long-term follow-up of a large series of patients prospectively managed with a “conservative” therapeutic strategy. JACC Cardiovasc Interv. (2012) 5(10):1062–70. 10.1016/j.jcin.2012.06.014 23078737

[27] Lettieri C Zavalloni D Rossini R Morici N Ettori F Leonzi O Management and long-term prognosis of spontaneous coronary artery dissection. Am J Cardiol. (2015) 116(1):66–73. 10.1016/j.amjcard.2015.03.039 25937347

[28] Rogowski S Maeder MT Weilenmann D Haager PK Ammann P Rohner F Spontaneous coronary artery dissection: angiographic follow-up and long-term clinical outcome in a predominantly medically treated population. Catheter Cardiovasc Interv. (2017) 89(1):59–68. 10.1002/ccd.26383 26708825

[29] Hassan S Samuel R Starovoytov A Lee C Aymong E Saw J. Outcomes of percutaneous coronary intervention in patients with spontaneous coronary artery dissection. J Interv Cardiol. (2021) 2021:6686230. 10.1155/2021/6686230 34104121 PMC8143895

[30] Chen S Merchant M Mahrer KN Lundstrom RJ Naderi S Goh AC. Spontaneous coronary artery dissection: clinical characteristics, management, and outcomes in a racially and ethnically diverse community-based cohort. Perm J. (2019) 23:18.278. 10.7812/TPP/18.278 31926571 PMC6836555

[31] Nakashima T Noguchi T Haruta S Yamamoto Y Oshima S Nakao K Prognostic impact of spontaneous coronary artery dissection in young female patients with acute myocardial infarction: a report from the angina pectoris-myocardial infarction multicenter investigators in Japan. Int J Cardiol. (2016) 207:341–8. 10.1016/j.ijcard.2016.01.188 26820364

[32] Mortensen KH Thuesen L Kristensen IB Christiansen EH. Spontaneous coronary artery dissection: a Western Denmark Heart Registry study. Catheter Cardiovasc Interv. (2009) 74(5):710–7. 10.1002/ccd.22115 19496145

[33] Olin JW Froehlich J Gu X Bacharach JM Eagle K Gray BH The United States registry for fibromuscular dysplasia: results in the first 447 patients. Circulation. (2012) 125(25):3182–90. 10.1161/CIRCULATIONAHA.112.091223 22615343

[34] Chen S Merchant M Mahrer KN Ambrosy AP Lundstrom RJ Naderi S. Pregnancy-associated spontaneous coronary artery dissection: clinical characteristics, outcomes, and risk during subsequent pregnancy. J Invasive Cardiol. (2021) 33(6):E457–66. 34001675 10.25270/jic/20.00529

[35] Clare R Duan L Phan D Moore N Jorgensen M Ichiuji A Characteristics and clinical outcomes of patients with spontaneous coronary artery dissection. J Am Heart Assoc. (2019) 8(10):e012570. 10.1161/JAHA.119.012570 31084345 PMC6585323

[36] Rashid HNZ Wong DTL Wijesekera H Gutman SJ Shanmugam VB Gulati R Incidence and characterisation of spontaneous coronary artery dissection as a cause of acute coronary syndrome—a single-centre Australian experience. Int J Cardiol. (2016) 202:336–8. 10.1016/j.ijcard.2015.09.072 26426273

[37] Cerrato E Giacobbe F Quadri G Macaya F Bianco M Mori R Antiplatelet therapy in patients with conservatively managed spontaneous coronary artery dissection from the multicentre DISCO registry. Eur Heart J. (2021) 42(33):3161–71. 10.1093/eurheartj/ehab372 34338759

[38] Alfonso F de la Torre Hernández JM Ibáñez B Sabaté M Pan M Gulati R Rationale and design of the BA-SCAD (beta-blockers and antiplatelet agents in patients with spontaneous coronary artery dissection) randomized clinical trial. Rev Esp Cardiol (Engl Ed). (2022) 75(6):515–22. 10.1016/j.recesp.2021.08.002 34561195

